# Driving new technologies in hospitals: association of organizational and personal factors with the readiness of neonatal intensive care unit staff toward webcam implementation

**DOI:** 10.1186/s12913-022-08072-5

**Published:** 2022-06-17

**Authors:** Jan Hoffmann, Alinda Reimer, Laura Mause, Andreas Müller, Neo-CamCare, Till Dresbach, Nadine Scholten

**Affiliations:** 1 University of Cologne, Faculty of Medicine and University Hospital Cologne, Faculty of Human Sciences, Institute for Medical Sociology, Health Services Research, and Rehabilitation Science (IMVR), Eupener Str. 129, 50933 Cologne, Germany; 2 Department of Neonatology and Pediatric Intensive Care Medicine, University Hospital Bonn, Venusberg-Campus 1, 53127 Bonn, Germany

**Keywords:** Neonatal intensive care units, Preterm, Webcams, Implementation, Technology, Technological innovation, Readiness, Acceptance, Germany

## Abstract

**Background:**

The use of webcam technology in neonatal intensive care units (NICUs) enables parents to see their child when the parents cannot be present at the NICU. The webcam’s use has been gaining increasing attention. Lead physicians and lead nursing staff play a key role in the decision of whether to implement webcams. This study investigates factors that are associated with the readiness for the implementation of a webcam system among lead NICU staff.

**Methods:**

A postal survey was conducted among all lead physicians and lead nursing staff in all German NICUs between December 2020 and April 2021 (total *N* = 416, one lead physician and one lead nursing staff per NICU, *N* = 208). On the basis of normalization process theory, personal (technology acceptance) and organizational (innovation climate) attributes were chosen to determine their association with the readiness for the implementation of a webcam system. The association of these factors was determined using multiple linear regression models for both lead physicians and lead nurses.

**Results:**

Overall, a response rate of 66.59% (*n* = 277) was achieved. Technology acceptance proved to be a significant factor associated with the readiness for the implementation of a webcam system among lead physicians. Furthermore, staff already working with webcams in their NICUs indicated a significantly higher level of technology acceptance than staff without webcam experience and without any desire to use a webcam in the future. No significant association was found between innovation climate and the readiness for the implementation of a webcam system.

**Conclusions:**

Technology acceptance was identified as a factor associated with the readiness for the implementation of a webcam system. The insights from this study can be used to manage potential barriers regarding the readiness for implementation of webcams in NICUs.

**Trial registration:**

The Neo-CamCare study is registered at the German Clinical Trials Register. DRKS-ID: DRKS00017755. Date of Registration in DRKS: 25-09-2019.

**Supplementary Information:**

The online version contains supplementary material available at 10.1186/s12913-022-08072-5.

## Background

The Organization for Economic Co-operation and Development defines health technology and innovation “as the application of knowledge to solve practical clinical and health problems, including products, procedures, and practice styles that alter the way health care is delivered” [1]. The use of webcams in neonatal intensive care units (NICUs) is one of the many technological innovations that have been implemented in hospitals. It enables parents of premature infants to virtually see their child at the times when they cannot be physically present in an NICU. Thus, the use of webcams helps to reduce stress levels in parents, strengthen the feelings of closeness to the child, and increase parental well-being. For parents, a premature birth is associated with high levels of stress [2], anxiety [3, 4], and depression [2, 3, 5]. Studies have indicated that webcams can positively influence parents’ health condition and the parent–child relationship [6–8].

However, implementing new technologies such as a webcam system in a hospital environment is challenging. The implementation of most new technologies leads to additional work and initially increases the staff’s workload and work-related stress levels among hospital staff, especially intensive care unit staff, are already high [9, 10].

The literature describes various theories regarding the implementation of new technologies and the concept of change caused by new technologies [11–16]; however, few are specific to health care organizations [15, 17]. The middle-range theory of normalization process theory was originally developed for the implementation of innovative health technologies [16–19]. A model for the implementation of innovative health technologies was developed, tested, and extended into a theory on the basis of qualitative and quantitative studies in the health care sector [17]. The process identified four constructs as being crucial for the successful implementation of innovations in health care organizations: coherence, cognitive participation, collective action, and reflexive monitoring [16, 17]. Consistent with the findings of Wanberg and Banas [14], who, in their research, focused on openness toward organizational change, the normalization process theory suggests that not only individual-specific variables but also context-specific factors influence the successful implementation of technologies.

For medical staff, webcams represent a new technology that needs to be implemented. The use of webcams may imply additional workload and changes in workflow owing to the assignment of webcam-related tasks and may negatively affect the ability to provide patient care [20]. Furthermore, the attitudes of staff toward a webcam system may influence its implementation. A recent study by Kubicka et al. showed that a majority of nurses believed that webcams increase parental and nursing stress. However, in the same study measurements showed that no significant differences in stress levels and burnout among staff resulted when webcams are used [21]. Further research from some NICUs that have already implemented a webcam system indicates more work disruptions caused by the need to adjust the webcams [20], privacy risks [7], and increased stress levels [20].

Uncertainty remains regarding the factors that may facilitate or hinder the readiness of lead staff with regard to the use of a webcam system prior to its implementation. In the context of this study, readiness for a webcam system entails a psychological aspect: the commitment toward the introduction of the system. If webcams were to be implemented in NICUs, the knowledge of these factors would be essential to target potential barriers to webcam implementation and address reservations regarding webcam use in NICUs.

### Study purpose

The results reported here are part of NeoCamCare, a publicly funded project that evaluates webcam use in NICUs and its advantages and disadvantages by considering the perspectives of parents and health care workers. The project aims to strengthen the evidence base for or against webcam use in NICUs [22].

The present study assesses the association of personal (technology acceptance) and organizational (innovation climate) factors on the readiness for the implementation of a webcam system in NICUs from the perspective of lead nurses and physicians.

## Methods

### Study design

A cross-sectional postal survey was conducted to investigate the association of organizational and personal factors on the readiness toward the implementation of webcam use in NICUs. To increase the response rate, three reminders were provided, following Dillman [23]. The first correspondence included a cover letter, the questionnaire, and a small incentive. The first reminder was sent in the form of a postcard to all participants a week after the initial correspondence, while the following two reminders were sent 3 and 7 weeks after the initial correspondence to participants who had not responded. These reminders included a cover letter and the questionnaire. All mail was sent by the Institute of Medical Sociology, Health Services Research, and Rehabilitation Science affiliated with the University of Cologne.

### Participants and sampling

The aim of the study was to conduct a survey with a lead nurse and a lead physician from every NICU in Germany. Thus, the inclusion criteria for study participation were to belong to the lead nursing or lead physician staff on German NICUs. A lead nurse and a lead physician from NICUs of all level 1 and 2 perinatal centers (equivalent to the highest perinatal care level) in Germany were invited to participate in the study. The level 1 and 2 perinatal centers were selected via a website [24] that serves as the official listing of perinatal centers in Germany. We collected mailing addresses for all participants from the hospitals’ websites. The initial sample identified from perinatalzentren.org consisted of 213 centers. As some hospitals listed on perinatalzentren.org cooperate with other hospitals and use the same facilities, the lead staff in NICUs were the same for those hospitals. Eventually, the lead staff of the NICUs of 208 perinatal centers were contacted.

For lead physicians, the senior physician in charge of the NICU was contacted. If the senior physician could not be identified from the website of a hospital, the chief physician of the pediatrics department was contacted. The questionnaire addressed the individual who could speak as a key physician for the NICU. Because most hospitals did not provide accurate information on the lead nursing staff of their NICUs on their websites, the lead nursing staff was addressed as “for the attention of the lead nurse.”

### Data collection

Data were collected between December 1, 2020, and March 31, 2021. Participants were asked to voluntarily return the completed questionnaire for this anonymous survey in a pre-stamped envelope.

### Questionnaire

**Fig. 1:**
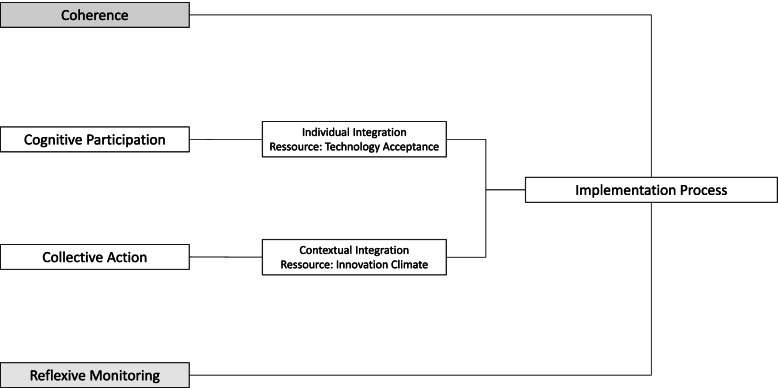
Adapted construct of the normalization process theory

The constructs of cognitive participation and collective action in the normalization process theory were operationalized by surveying individual and context variables (Fig. 1). A questionnaire consisting of validated scales as well as self-developed items was used to assess personal attributes, organizational attributes, and readiness for the implementation of a webcam system. Cognitive participation was operationalized through the construct of technology acceptance, whereas collective action was operationalized through the construct of innovation climate.

#### Measures

##### Dependent variable

###### Readiness for change 

To assess the readiness for the implementation of a webcam system in the NICU, we used the validated Readiness for Change scale [15]. The scale is part of the Organizational Change Questionnaire developed by Bouckenooghe et al. [15]. The questionnaire measures readiness for change in organizations and is used to support the implementation process of new technologies in health care organizations. The instrument has been validated in four studies [15]. In this survey, the constructs of intentional readiness for change and emotional readiness for change were used. In the English version of the Organizational Change Questionnaire, the third item (“I find the change refreshing”) from the Emotional Readiness for Change scale was omitted to achieve an adequate fit for a three-factor model. In this survey, we also omitted this item [15]. In the validation study, the constructs of intentional readiness for change and emotional readiness for change achieved satisfactory psychometric properties (Cronbach’s alpha; intentional readiness for change: 0.89; emotional readiness for change: 0.70) [15]. In this study, we combined the intentional readiness and emotional readiness items into one scale that was used to determine NICU staff’s willingness to implement a webcam system in their wards. The scale consists of five items answered on a 5-point Likert scale ranging from 1 (strongly disagree) to 5 (strongly agree).

Because no German version of the Organizational Change Questionnaire was available, in agreement with the authors of the original scale, the constructs intentional readiness for change and emotional readiness for change were translated into German following the TRAPD approach [25] and adapted to the webcam technology. The TRAPD approach entails 5-steps (translation, review, adjudication, pretest, and documentation). The translation was conducted by two independent translators (professional translator and research staff). A discussion about the translation was held between the two translators and JH. Adjudication was achieved by NS, LM, and AR. The wording of all items used in the survey can be found in Additional file 1.

##### Independent variables

###### Innovation climate

To assess the innovation climate in NICUs from the perspective of staff, the validated Innovation Climate scale from the German version of the team climate inventory was used [26]. The scale consists of eight items measured on a 5-point Likert scale ranging from 1 (not true at all) to 5 (entirely true). The construct of innovation climate achieved good psychometric values in the German validation study (Cronbach’s alpha 0.87) [26]. The wording of all items used can be found in Additional file 2.

###### Technology acceptance

To measure technology acceptance for the webcam system, the subscale technology acceptance from the validated Technology Commitment scale by Neyer et al. was used [27]. The scale technology acceptance, which consisted of four items was measured on a 5-point Likert scale ranging from 1 (not true at all) to 5 (entirely true). The construct of technology acceptance showed good psychometric properties (Cronbach’s alpha 0.83) [27]. The wording of all items used can be found in Additional file 3.

### Data analysis

RStudio (version 1.4.1106) was used for all data analyses. To analyze the association of innovation climate and technology acceptance with the readiness of staff regarding the implementation of webcams, a multiple linear regression model was used. For all constructs, average scale values were calculated by summing up single item values and dividing them by the number of items per scale. Cases were excluded if they had two or more missing values per scale. For the Innovation Climate scale, we imputed missing values based on the mode of each item for this scale following the instructions provided by the scale’s authors. Additionally, the categorical variables age and gender were added as control variables. After the calculation of average scale values, only complete cases were included in the regression analysis. Before the regression models were estimated, independent variables were checked for outliers, multicollinearity, homoscedasticity, multivariate normality, and linearity. The generalized variance inflation factor was used to determine multicollinearity. In case of multicollinearity, items were removed from the model. For all significance tests, we used an alpha level of 0.05. To control for multiple testing, the Bonferroni correction was used. For each model, the Akaike information criterion (AIC) was used to determine the model’s goodness of fit. Metric variables were checked for normal distribution using Shapiro–Wilk test. If variables were not normally distributed, median and interquartile range (IQR) are reported. We calculated an intercept-only model as the reference model for goodness of fit. Characteristics such as gender and age differ between physicians and nursing staff in German hospitals [28], as a majority of physicians are older and male, in contrast with the majority of nursing staff, who tend to be younger and female. Therefore, models were estimated separately for these groups.

Participants who already used a webcam in their ward were identified via one single item (“Are webcams currently in use in your ward?”). These participants were excluded from the regression analysis as webcams have already been implemented in the ward. After regression analysis, a separate group comparison was performed to investigate differences in technology acceptance and innovation climate between participants who already used a webcam system in their NICU and participants who did not wish to use a webcam system. One self-developed item with the wording “Would you like to introduce the webcam system to your ward?” was used to identify participants who did not wish to use a webcam system. The Mann–Whitney U test was used to determine significant differences.

To control for a type 2 error rate, we performed a post-hoc power analysis given alpha, sample size, and effect size for all analysis. For power calculation, we used the latest version of GPower 3.1.9.7.

The reporting of this study adheres to the STROBE statement for reporting observational studies [29]. A completed checklist of reported items can be found in Additional file 4.

## Results

Of the study population, 277 of 416 participants completed and returned the questionnaire, for a response rate of 66.59% (145 physicians [69.71%]; 132 nurses [63.46%]). Of these, 33 participants stated that they already used a webcam system in their ward. These participants were excluded from the regression analysis. In the sample of participants who had not used a webcam system before, 10 cases were removed owing to missing values; thus, 234 cases were used in the regression analysis. In the sample of participants who indicated prior webcam use in their ward, two cases were removed owing to missing values, and the data of 31 participants were used for the group comparison. Variable values did not change significantly after the removal of missing values. The characteristics of the study participants in both the regression analysis and the group comparison are displayed in Table 1.

**Table 1:** Characteristics of 265 study participants

	Sample set for regression analysis		Sample set for group comparison	
Variable	Physicians, *N* = 122	Nurses, *N* = 112	Participants with webcam use, *N* = 31	Participants without webcam use and no desire to use one, *N* = 54
Age, n (%)				
≤44 years	30 (24.59%)	40 (35.71%)	6 (19.35%)	11 (20.37%)
45–54 years	48 (39.34%)	48 (42.86%)	15 (48.39%)	26 (48.15%)
≥55 years	44 (36.07%)	24 (21.43%)	10 (32.26%)	17 (31.48%)
Gender, n (%)				
Male	88 (72.13%)	7 (6.25%)	15 (48.39%)	23 (42.59%)
Female	34 (27.87%)	105 (93.75%)	16 (51.61%)	31 (57.41%)
Readiness for change (IQR)	3.00 (2.20–4.00)	3.00 (2.40–3.80)		
Technology acceptance, median (IQR)	3.75 (3.25–4.00)	3.75 (3.25–4.25)	4.00 (3.50–4.25)	3.50 (3.00–3.94)
Innovation climate, median (IQR)	3.50 (3.25–3.75)	3.62 (3.25–3.88)	3.62 (3.25–3.81)	3.62 (3.16–3.75)

*n* = 265; *IQR* interquartile range

In the regression sample, the majority of physicians were aged 45 years or older (75.41%). Nursing staff showed a slightly younger age distribution, with 35.71% of participants being aged 44 years or younger and 21.43% being aged above 55 years. Regarding gender distribution, 72.13% of physicians and 6.25% of nursing staff identified as male.

The Readiness for Change, Technology Acceptance, and Innovation Climate scales showed good psychometrics properties in our sample with standardized Cronbach’s alpha of 0.95, 0.83, and 0.85, respectively.

**Fig. 2:**
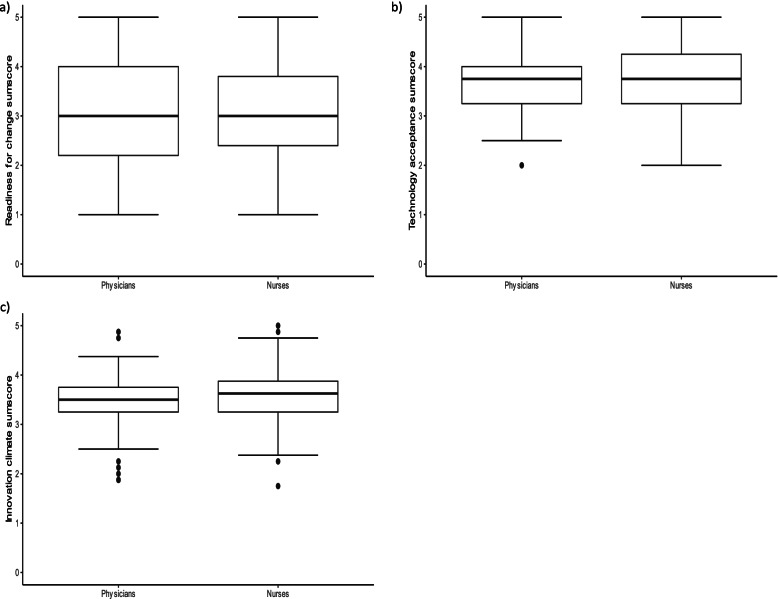
Boxplots of dependent and independent metric variables

Differences in the median scale value for the subgroup of physicians and nurses were detected only on the Innovation Climate scale. Here, nursing staff scored slightly higher than physicians (3.62 vs. 3.50). For physicians and nurses, the median value for the dependent variable readiness for change was 3 on a scale from 1 to 5, with 50% of physicians scoring between 2.2 and 4.0 (between 2.4 and 3.8 for nurses). Figure 2 illustrates the distribution of dependent and independent metric variables.

We calculated four multiple linear regression models for each subgroup: an intercept model, a model that controlled for age but not for gender, a model including an interaction between technology acceptance and age, and a model controlling for age and gender. Interaction effects were dismissed because they were not statistically significant and did not improve the goodness of fit of the models. The final model was selected according to the criteria of low AIC and high adjusted R^2^. Table 2 shows the final models for physicians and nursing staff.

**Table 2:** Multiple linear regression with 122 physicians and 112 nurses

**Physicians**						
Characteristic	Beta	SE^a^	95% CI^b^	*P*-value^c^	GVIF^d^	Adjusted GVIF^e^
(Intercept)	2.84	0.872	1.11–4.57	0.009		
Technology acceptance	0.38	0.140	0.10–0.65	0.049	1.1	1.0
Innovation climate	−0.23	0.186	−0.60–0.14	> 0.9	1.0	1.0
Age					1.1	1.0
≤44 years	–	–	–			
45–54 years	−0.46	0.237	−0.93–0.01	0.3		
≥55 years	−0.34	0.249	−0.83–0.16	> 0.9		
Gender					1.0	1.0
Male	–	–	–			
Female	−0.27	0.208	−0.68–0.15	> 0.9		
R² = 0.118; Adjusted R² = 0.080; Sigma = 1.01; Statistic = 3.11; *P*-value = 0.011; df = 5; Log-likelihood = -172; AIC = 357; BIC = 377; Deviance = 119; Residual df = 116; *n* = 122, Power (1 – β error probability) = 0.91						
**Nursing staff**						
Characteristic	Beta	SE^a^	95% CI^a^	*P*-value^b^	GVIF^a^	Adjusted GVIF^ab^
(Intercept)	2.87	0.762	1.35–4.38	0.002		
Technology acceptance	0.03	0.140	−0.25–0.31	> 0.9	1.3	1.2
Innovation climate	0.10	0.183	−0.26–0.46	> 0.9	1.4	1.2
Age					1.2	1.0
≤ 44 years	–	–	–			
45–54 years	−0.46	0.216	−0.89–-0.03	0.2		
≥ 55 years	0.11	0.255	−0.40–0.61	> 0.9		
Gender					1.0	1.0
Male	–	–	–			
Female	−0.13	0.375	−0.87–0.62	> 0.9		
R² = 0.073; Adjusted R² = 0.029; Sigma = 0.939; Statistic = 1.67; *P*-value = 0.15; df = 5; Log-likelihood = -149; AIC = 312; BIC = 331; Deviance = 93.4; Residual df = 106; *n* = 112, Power (1 – β error probability) = 0.63						

^a^ SE = standard error, ^b^CI = confidence interval, ^c^Bonferroni correction for multiple testing, ^d^ GVIF = generalized variance inflation factor, ^e^GVIF^[1/(2*df)]

For the group of physicians, 122 participants were included in the regression analysis. As shown in Table 2, in our sample of physicians, a higher technology acceptance was positively associated (*P*-value: 0.049) with the readiness for the implementation of a webcam system (Beta = 0.38), whereas innovation climate, age, and gender were not significantly associated with the readiness for the implementation of a webcam system.

Of our sample of nurses, 112 participants were included in the regression analysis. Technology acceptance and innovation climate were not significantly associated with the readiness for the implementation of a webcam system. In line with the findings for the physician subgroup, age and gender were not significantly associated with the readiness for a webcam system either.

The group comparison of study participants with prior webcam experience and study participants without prior webcam experience and no wish to use a webcam in the future showed the following: Technology acceptance was significantly higher among study participants with prior webcam experience than in the group of study participants without prior webcam experience (*P*-value: 0.007). With a medium effect size (Cohen’s d = 0.57) the power of this test amounted to 0.69. There was no significant difference in innovation climate between these groups (*P*-value: 0.696, Cohen’s d = 0.015, Power = 0.05).

## Discussion

The present study was designed to determine the association between the constructs technology acceptance and innovation climate and the readiness for the implementation of a webcam system among physicians and nursing staff in German NICUs. The analysis leads to the following conclusions: First, technology acceptance significantly associates with the readiness for the implementation of a webcam system among physicians in German NICUs. Second, physicians and nursing staff who were already using a webcam system in their wards had a significantly higher technology acceptance than staff who did not wish to use a webcam system in the future. Both findings demonstrate the impact of technology acceptance on the implementation of new technologies such as webcams in NICUs. However, this study did not find a significant effect of technology acceptance among nursing staff. Surprisingly, innovation climate did not show a significant association with the readiness for the implementation of a webcam system. An explanation might be a difference between innovation climate as an organizational construct and the personal readiness for the implementation of a webcam system. In both physicians and nurses, age was not significantly associated with the readiness for the implementation of a webcam system. The age and gender distribution of our subgroups proved to be typical for a German hospital setting: physicians tended to be older and male, whereas nurses were younger and female [28]. Regarding the distribution of the dependent variable readiness for the implementation of a webcam system, the boxplots show a large IQR and an aggregation of values around the scale value 3, indicating that a proportion of the study participants in both groups showed a moderate readiness for the implementation of a webcam system. This finding is consistent with that of Hennemann et al., who found that almost half of the health care professionals in their study sample showed only moderate acceptance of eHealth Interventions [30]. Hawkes et al. found that the privacy risks or personal stress associated with webcam use are additional factors influencing the attitude toward the implementation of a webcam system among health care professionals [7]. It is noteworthy that the study participants, who’s data entered regression analysis, had not used a webcam system in the past or at the time of the survey. Although the questionnaire contained information on how a webcam system functions in the NICU setting, a lack of education about such a system may have influenced the participants’ readiness for the implementation of a webcam system. In a study among health care professionals Le Bris et al. found that aspects associated with live video emerged, such as the impact on parents, benefits for newborns, or the impact of video recording on healthcare professionals’ behavior [31]. These aspects may also play a role when it comes to the decision of whether to introduce a webcam system. However, the present study focused on personal attributes such as technology acceptance and innovation climate as an organizational attribute and their influence on the readiness for a webcam system in NICUs.

### Strength and limitations

We sent the questionnaire to all NICUs in Germany in an attempt to capture as many opinions on the topic of the use of webcams in NICUs as possible. Prior studies have shown that response rates among German health care professionals, especially in inpatient care, are low. Sturm et al. reported a response rate of 37% among physicians and 39% among nurses in their study, whereas Raspe et al. reported a response rate of 13% among young physicians and nurses in German hospitals [10, 32]. In our study, 277 of 416 participants completed and returned a questionnaire, for a response rate of 66.59%.

However, when performing subgroup analysis, the sample size remained small, which may potentially have impacted the regression models and *P*-values. For physicians, our regression model explained 8% of the variance after the adjustment of R^2^; for nurses, only 2.9% of variance could be explained after adjustment. Yet, we decided not to include additional variables in the model because we aimed to verify our theory-driven hypothesis that technology acceptance and innovation climate are associated with the readiness for the implementation of a webcam system among physicians and nurses. Although the model for physicians showed a significant effect of technology acceptance on the readiness for the implementation of a webcam system, there seem to be other factors that influence such readiness among physicians and nursing staff. For instance, concerns regarding privacy risks [7] and the impact of video recording on health care professionals’ behavior [31] may also influence the readiness for the implementation of a webcam system. Furthermore, physicians and nursing staff may be concerned that the use of webcams only facilitates a one-way connection from parent to child, as opposed to interaction between parents and child, and that webcam use may reduce the number of parental visits.

## Conclusion

Multiple regression analysis revealed that only for lead physicians, technology acceptance was significantly associated with the readiness for the implementation of a webcam system. Leading NICU staff who already used a webcam system in their wards show a significantly higher technology acceptance than lead NICU staff who had not used a webcam system previously and did not wish to do so in the future. Regarding the implementation of webcams in NICUs, technology acceptance of staff should be considered, and reservations should be addressed with appropriate training and information.

## Supplementary Information

**Additional file 1.** Readiness for Change Scale. Wording of Readiness for Change Scale items used in the questionnaire.

**Additional file 2.** Innovation Climate Scale. Wording of Innovation Climate Scale items used in the questionnaire.

**Additional file 3.** Technology Acceptance Scale. Wording of Technology Acceptance Scale items used in the questionnaire.

**Additional file 4.** STROBE checklist for cross-sectional studies. Completed checklist for Items to be reported in cross-sectional studies.

**Additional file 5.** List of consortium members. Full list of Neo-CamCare applicants.

## Data Availability

The datasets generated and analyzed during the current study are not publicly available due to Ethical restrictions by the Ethics Committee of the Medical Faculty of the University of Cologne in order to protect participant confidentiality. However, data are available from the corresponding author on reasonable request.

## Abbreviations

IMVR: Institute for Medical Sociology, Health Services Research, and Rehabilitation Science
NICU: Neonatal intensive care unit
AIC: Akaike information criterion
IQR: Interquartile range
