# Supplementary material for: Driving new technologies in hospitals: association of organizational and personal factors with the readiness of neonatal intensive care unit staff toward webcam implementation
Source: BMC Health Serv Res. 2022 Jun 17;22:787. doi: 10.1186/s12913-022-08072-5 (PMC9205038; doi:10.1186/s12913-022-08072-5)
Supplement: Supplementary file 1 — Additional file 1. Readiness for Change Scale. Wording of Readiness for Change Scale items used in the questionnaire. [file 12913_2022_8072_MOESM1_ESM.pdf]

Readiness for Change Scale

	Strongly disagree	Rather disagree	Neither	Rather agree	Strongly agree
I consider the introduction of webcams in neonatological intensive care units is basically the right step.	☐	☐	☐	☐	☐
I am ready to play a key role in the introduction of the webcams.	☐	☐	☐	☐	☐
I am ready to make an extra effort for the introduction of the webcams.	☐	☐	☐	☐	☐
I have a good feeling about the use of webcams on our ward.	☐	☐	☐	☐	☐
I would see the use of webcams as a positive development on our ward.	☐	☐	☐	☐	☐
