# Supplementary material for: Driving new technologies in hospitals: association of organizational and personal factors with the readiness of neonatal intensive care unit staff toward webcam implementation
Source: BMC Health Serv Res. 2022 Jun 17;22:787. doi: 10.1186/s12913-022-08072-5 (PMC9205038; doi:10.1186/s12913-022-08072-5)
Supplement: Supplementary file 2 — Additional file 2. Innovation Climate Scale. Wording of Innovation Climate Scale items used in the questionnaire. [file 12913_2022_8072_MOESM2_ESM.pdf]

**Innovation Climate Scale**

| | Not true at all | Rather not true | Partly true | Rather true | Entirely true |
|---|---|---|---|---|---|
| We are promptly and readily supported in the development of new ideas. | ☐ | ☐ | ☐ | ☐ | ☐ |
| The team is open and receptive to change. | ☐ | ☐ | ☐ | ☐ | ☐ |
| People on the team are constantly looking for new ways to look at problems. | ☐ | ☐ | ☐ | ☐ | ☐ |
| The team is constantly moving towards the development of new answers. | ☐ | ☐ | ☐ | ☐ | ☐ |
| In our team, we take the time we need to develop new ideas. | ☐ | ☐ | ☐ | ☐ | ☐ |
| People on the team work together to develop and implement new ideas. | ☐ | ☐ | ☐ | ☐ | ☐ |
| Team members provide and willingly share resources to help realize new ideas. | ☐ | ☐ | ☐ | ☐ | ☐ |
| Team members provide practical support for new ideas and their realization. | ☐ | ☐ | ☐ | ☐ | ☐ |
