# Supplementary material for: Driving new technologies in hospitals: association of organizational and personal factors with the readiness of neonatal intensive care unit staff toward webcam implementation
Source: BMC Health Serv Res. 2022 Jun 17;22:787. doi: 10.1186/s12913-022-08072-5 (PMC9205038; doi:10.1186/s12913-022-08072-5)
Supplement: Supplementary file 3 — Additional file 3. Technology Acceptance Scale. Wording of Technology Acceptance Scale items used in the questionnaire. [file 12913_2022_8072_MOESM3_ESM.pdf]

Technology Acceptance Scale

	Not true at all	Rather not true	Partly true	Rather true	Entirely true
I am very curious about new technical developments.	☐	☐	☐	☐	☐
I quickly take a liking to new technical developments.	☐	☐	☐	☐	☐
I am always interested in using the latest technical equipment.	☐	☐	☐	☐	☐
If I had the opportunity, I would use use technical products much more often than I do at present.	☐	☐	☐	☐	☐
