# Supplementary material for: Driving new technologies in hospitals: association of organizational and personal factors with the readiness of neonatal intensive care unit staff toward webcam implementation
Source: BMC Health Serv Res. 2022 Jun 17;22:787. doi: 10.1186/s12913-022-08072-5 (PMC9205038; doi:10.1186/s12913-022-08072-5)
Supplement: Supplementary file 5 — Additional file 5. List of consortium members. Full list of Neo-CamCare applicants. [file 12913_2022_8072_MOESM5_ESM.pdf]

List of consortium members

Neo-CamCare

Dr. Nadine Scholten[1], Prof. Dr. Andreas Müller[2], Dr. Till Dresbach[2], Prof. Dr. Martin Hellmich[3], Christina Samel[3], Prof. Dr. Christiane Woopen[4], Christiane Jannes[5], Prof. Dr. Ludwig Kuntz[6], Prof. Dr. Indra Spiecker gen. Döhmann[7], Dr. Sebastian Bretthauer[7], Dr. Dirk Horenkamp-Sonntag[8], Stefanie Wobbe-Ribinski[9]

[3]Institute of Medical Statistics and Computational Biology, University Hospital Cologne, Cologne, Germany. [4]Center for Life Ethics, University of Bonn, Bonn, Germany. [5]Cologne center for ethics, rights, economics, and social sciences of health, University of Cologne, Cologne, Germany. [6]Department of Business Administration and Health Care Management, University of Cologne, Cologne, Germany. [7]Data Protection Research Unit, Goethe-University of Frankfurt, Frankfurt, Germany. [8]Techniker Krankenkasse, Hamburg, Germany. [9]DAK Gesundheit, Hamburg, Germany.
